# On Dissection-Wounds

**Published:** 1830-04-01

**Authors:** 


### XLII.

### ON DISSECTION-WOUNDS. By WM. LAWRENCE, Esq.

In the notes of Mr. Lawrence's lectures, as published in the Medical Gazette, the following interesting section on wounds received during the dissection of human bodies, is transferred to our pages because the subject is of great importance—the writer a man of high authority—and the doctrines and practical precepts somewhat doubtful. The document itself shall be fairly laid before our readers, ere we make any comment. We have taken the liberty, however, of marking some passages in Italics, as they will arrest the reader's attention more strongly and render reference more easy.

### WOUNDS RECEIVED IN DISSECTION.

"In the division of poisoned wounds I have marked down in the syllabus of these lectures injuries received in dissection?—with a query, a note of interrogation; and I do this in order to express the doubt which I feel in my own mind, whether the effects of such injuries be owing to the introduction into the human frame of a poison or not. *It seems to me very doubtful in this case, whether any thing that can be called virulent or poisonous is introduced into the human frame by these occurrences, or whether the effects are to be explained as resulting from such injuries considered mechanically.*

If these be poisoned wounds, they certainly follow other laws from what we observe in those cases of poison that we are intimately acquainted with. If we look at the small-pox, cow-pox, scarlet fever, or syphilis, we see that the application of poison produces pretty regularly certain effects; that they will take place within a certain time, that they exhibit a certain character, and that you can before-hand ascertain pretty clearly, the symptoms and course of such injuries. But we can give no such description of the injuries that arise from dissection; —if they arise, therefore, from poison, it is one of an uncertain, and we might almost say, capricious kind. In the first place, in a great majority of instances, no injurious effect is produced from wounds received in dissection;—there are hundreds and hundreds of these wounds occurring without any injurious consequence. It is only in a very small proportion out of the whole number of such wounds, that any prejudicial effects are produced upon the human frame. We can, perhaps, quite as well explain the occurrence of these effects when they take place, by the state of the health of the individual in whom the phenomena occur, as by any peculiar virulent property that may be applied in these cases. Now it has happened to me to meet with cases where wounds have taken place in dissection, and where a person has cut himself hundreds and hundreds of times when he has been in a healthy state of body, who has afterwards died under a like disease. I never experienced any ill effects but once, and then I was in a bad state of health. I had an inflammation in the finger, and subsequently a swelling of the glands in the axilla, with induration. There are a certain number of cases—but very few compared with the whole—in which undoubtedly serious local effects are produced, and in which serious general symptoms occur.

It is, perhaps, rather a question of curiosity than one of direct practical consequence, whether these effects arise from a poisonous matter communicated to the part, or whether they owe their origin to the particular state of the individual at the time the wound is inflicted.

In the first place, we cannot point out any particular state of a subject, or any condition of previous disease, that will certainly give rise to any sort of symptoms in these cases. Indeed we shall see that an individual gets a prick or a cut in the dissection of a certain subject, and suffers serious consequences from it, while others, who have had to do with the same subject, suffer no injurious consequences at all. Thus in the majority of instances, the effects produced are such as would seem to arise in wounds considered in themselves, without any reference to the virulent state or decomposition of the bodies in the dissection of which they occur. Inflammation comes on in the part that is the seat of the wound; suppuration may take place; the absorbents may become inflamed, and the absorbent glands, from which these vessels lead, may participate in the inflammation; the cellular substance of the part also becomes the subject of inflammation; and thus, perhaps, it is seen generally, that phlegmonous erysipelas is produced. This condition is a serious one; it is capable in itself, without any suspicion of poisonous properties in the cause that produced it, to give rise to very serious local, and equally serious general symptoms. Thus a great majority of the cases in which serious symptoms arise, admit of explanation on ordinary principles without the suspicion of any poisonous property in the immediate cause. The question, therefore, respecting the existence of poison, is confined to a few cases, in which some particular local or general symptoms are produced.

With respect to a great number of the ordinary cases, I think there can be no doubt in referring the phenomena they exhibit merely to the effects of the wound, considered as a cause of local inflammation. There was a gentleman, formerly a pupil of this hospital, who wounded his thumb in sewing up a body. *It was the body of a female, who died of some disease in the peritoneum, and I believe he was hardly aware of having injured himself. However, in the course of the night after he received this injury, he experienced very severe pain in the part, (he might have scratched himself slightly, but he felt nothing till night) and became extremely unwell. When he awoke in the morning he sent for a medical friend, who found him in a state of great excitement. He was a robust and hearty person of full habit.* His friend found him with a full, hard, and strong pulse, and with considerable swelling about the part in which the injury had been received—a swelling extending from his hand to the fore-arm generally. He was in a state of extraordinary agitation and restlessness—his nervous system was so much disturbed that he could hardly keep himself quiet. He was in a state, in fact, which called for active depletion; it was adopted, and he lost thirty ounces of blood with considerable relief. He was better the next day, but still the upper extremity generally was swelled. The absorbents leading from the thumb along the fore-arm, and the absorbent glands in the axilla, became inflamed. He had pain in the head, and the nervous symptoms continued to a great degree. He had leeches applied to the head, and cold to the part injured, and purgative medicines were administered. On the following day, all the symptoms were worse; the limb was more swelled; the inflammation of the absorbents and glands was more obvious, and all the symptoms more severe. I saw him on this day; he fancied from the swelling of the ball of the thumb on which the injury was received, that there must be matter, and he wished it to be let out. A deep incision was made, and a little matter did flow out. The hand was enveloped in a warm poultice, and he received considerable relief. Now the incision was deep, and when the limb was enveloped in the poultice, he lost, without being sensible of it, thirty ounces of blood, and seemed better in consequence of this loss. However, the swelling of the fore-arm and upper-arm continued, and rather increased, while the nervous symptoms went on in a greater or less degree. On the following day, in consequence of the continuance of these symptoms, he lost blood twice, and had a quantity of leeches applied to the hand, fore-arm and upper-arm;—indeed he found that was the only way in which the excessive suffering and tension of the inflamed tumefaction could be relieved; so that, without knowing their quantity, he took a handful of leeches, and when they dropped off he put on another—and in the course of twenty-four hours 200 leeches were applied to the upper extremity. By this means the inflammatory action was pretty effectually reduced, and after three or four days of this treatment he found himself exceedingly exhausted. Mr. Gordon was with him, and a remarkable change took place in the symptoms. He became pallid in the countenance, cold in the extremities, the action of the heart was so enfeebled that he appeared as though he were about to die. Under these circumstances, Mr. Gordon gave him opium, which relieved him; he then continued exhibiting opium till the symptoms were removed;—and under the exhibition of the medicine in this way, he gradually recovered. Now in this case we can see nothing more than a local effect, producing high inflammatory action in an individual whose constitutional derangement may have occasioned that disturbance. We see in the treatment depletion, with the loss of a great quantity of blood, locally and generally, and the effect of this in controlling the inflammation. In this case we do not want the action of poison to explain the symptoms that occurred in it.

There are other instances in which the general and local disturbances have been different from the above; and it is in them principally that the explanation has been adduced, by which the agency of poison is supposed to be concerned in these cases.

There was a physician in the neighbourhood of London who examined the body of a woman that had died from puerperal peritonitis. At 8 o'clock on the morning of the 28th of December he assisted in sewing up the body, *and he was not aware that he had injured himself.** At 8 o'clock on the evening of the same day, being then dining in company with a friend, he felt a stinging heat and uneasiness at the end of one of his fingers, and he thought he might have wounded himself. On looking at his finger, a slight blush was observed; and when the part was examined, a slight opening was perceived, so that the inference was, that he had injured that part of his finger in sewing up the body. He thought he would try nitrate of silver, and he also put upon it a small quantity of nitric acid, that having been his habit as a matter of precaution. These applications were unattended with pain. He went home, and finding the finger still uneasy, and as the former applications had not given him any pain, he again applied nitrate of silver to the part, continuing the application till he felt it sensibly. The pain thus produced soon increased to a high degree of agony. Shivering came on, and he passed a restless and turbulent night; and when he was seen early in the morning, red lines had formed along the back of the hand. At 8 o'clock on the morning of the 29th, (he had opened the body at 8 o'clock on the 28th of Dec.) an eschar was observed the size of a pea, which was supposed to have occurred from the nitrate of silver. Leeches were directed for him, fomentations, and aperient medicines. About 1 o'clock on the same day, that is, the day after that on which he had opened the body, the finger in question seemed swelled, with a livid appearance; and the pain being very considerable, his medical friend, who saw him, made an incision through the integuments down to the bone, and by so doing he found the two last joints of the finger had mortified. The last and middle phalanx of the finger were already in a state of gangrene;—red lines were formed along the back of the hand and arm up to the elbow, and uneasiness was felt in the axilla. At this time he experienced complete prostration of strength; he felt himself as weak as a child. There was irregularity of his breathing; a sort of torpor about his arm; his pulse from 90 to 100, and soft. During the rest of the day he had much heavy kind of sleep, with intervals of severe pain; the hand and arm swelled, but not very considerably. The absorbents inflamed along the hand, and the axillary glands swelled, and great torpor was experienced, with difficulty of breathing. Swelling took place in the axilla and at the side of the chest, and openings were made in those situations without giving vent to any matter. He died at 6 o'clock on the morning of the 1st of January, which was on the fifth day after opening the body. Now, in this case, there is a remarkable local effect produced; that is, mortification in the part on which the injury had been received—and a serious influence exerted on the animal economy, by which, in four days, death is produced in an individual previously healthy.

A gentleman, a few years ago, who was a dresser at this hospital, opened a patient in the course of the day. He was not very exemplary, I believe, as to his mode of living, but indulged in the pleasures of the table; in short, not quite a pattern as to regularity. He got merely a prick on one of his fingers. On the same day that this took place, he had a large party of friends at his house, and he drank very freely. In the course of the night he was awoke by excessive pain in his finger, and before the middle of the following day, the last phalanx of the member had mortified. There was a swelling of that part of the hand and of the limb generally. Inflammation of the absorbents and the absorbent glands took place in this gentleman, with considerable fever. Subsequently, general inflammation of the skin and cellular membrane, that is, phlegmonous erysipelas of the hand and fore-arm, occurred. He was in a state of great danger, but by making a large incision through the inflamed part of the skin and the cellular membrane, he recovered.

Now it must be observed in the first of these two cases, that of the physician who examined the body and died in four days, and in many other of the most serious cases that have occurred, the in-

* Dr. Pett (we should suppose.)

juries have been received in the examination of patients who have died from inflammation of the peritoneum, and more particularly from puerperal peritonitis, so that if a poisonous influence is communicated to the body, it would seem to be most generally produced in instances of that kind. Here we have the conflicting result of these two cases. We have the instance of one individual in whom mortification takes place at an early period, as the result of injury, who dies; and another instance in which mortification occurs, and recovery takes place.

Now as to the occurrence of mortification consequent on the wound, I do not deem it to be a sufficient proof of the application of poison. I remember a butcher's boy who was brought to this hospital and placed under my care, who had a hook stuck in his hand, and which tore out its way so as to make a triangular flap on the palm of the hand—a sort of flap that we entertained no doubt would, by keeping it down, unite with the subjacent parts. But the flap mortified, although the injury had been produced merely by an iron hook; so that the mere consequence of a wounded part going into a state of mortification does not prove that poisonous influence is exerted, nor does it appear to me that in this case the general system exhibited the peculiarity that leads us to infer that poisonous influence took place. We merely see in this case that sympathetic influence of the circulating and nervous systems which may be produced by inflammation in a particular state of health, which in one individual will terminate fatally, and in another recovery will take place: so that we have no sufficient ground in any of these cases that poison is communicated to the frame, and from the evidence now before the public, I remain in doubt as to whether there is any poison in the case or not.

I am aware that animal substances in certain states of decomposition, are capable of producing a directly deleterious influence on the human frame. I have already had occasion to mention, in speaking of mortification and diseases of that kind, such disturbances as malignant pustule, where mortification of the surface takes place. This is a kind of effect not so often seen in this country as in some others, where it is observed among the butchers, who have the flaying and cutting-up of animals in a putrid state, which exerts this influence often to a fatal degree. That particular effect is described more minutely by Professor Delpech, in a work, entitled "Treatment of Surgical Diseases." We have not much opportunity of seeing it in this country, but in those instances there is a certain form the disease takes—a particular course which points out the operation of certain and peculiar causes; but we do not see this regularity in those serious occurrences which occasionally arise from dissection.

Now, with respect to the practical rules for the management of these injuries, some persons adopt the plan of touching any wound of this kind with nitrate of silver. I should suppose it is a safe and unobjectionable mode of proceeding, and that in the case of a slight wound, or puncture in dissection, there can be no harm in washing the part and touching it with nitrate of silver, which is likely to destroy any injurious influence that might otherwise take place. Some have recommended washing the surface of the wound with oil of turpentine, which might have a similar result. These are means of a preventive kind. If any inflammation should come on, then I conceive it would be necessary to keep the wounded part at rest, and to foment or poultice it—that is, to apply a soothing application to it. If there were symptoms of decided inflammation, to take blood from the part by leeches, to take means to evacuate the alimentary canal, and to pursue those measures until the danger should have gone by. If more considerable inflammation should have come on, and if matter should have formed, then I should consider it advisable to open any such collection of matter freely. In those cases where inflammation, swelling, and any thing like the formation of matter should occur, in addition to that in the seat of injury—that is, for example, in cases of a wound of the finger or hand, where redness and swelling occurs about the axilla or chest, if any thing like the formation of matter should be observed, I think the best course of proceeding would be freely to open the part. The danger in this case is of the inflammation increasing and spreading to the cellular membrane of such parts. When it does so, we know very well that there is a want of tendency to limit the inflammation; that such inflammation is apt to creep on, and affect the surrounding parts to a great extent; that it does not limit itself to one circumference; that it does not tend to come to the surface, and therefore a free incision throughout the affected part is, according to present experience, the most advantageous mode of treating such occurrences.

As to the *constitutional* disturbance that may ensue in conjunction with these local symptoms, generally speaking, it is of an inflammatory character; and it must be treated by antiphlogistic means, according to the extent of the disturbance.

On the whole, I confess I do not regard these cases with any thing like the feelings of alarm that some persons do. In a great majority of instances, if a proper degree of attention is paid, they terminate very favorably. I do not conceive that, generally speaking, they are cases that should give rise to alarm, or be looked upon with apprehension. I acknowledge that I am rather inclined to discourage, as much as I can, the idea of a poisonous nature attached to these wounds, because I conceive that the opinion produces much alarm. I do not, however, argue against their poisonous nature from this notion, but I give you my opinion formed from a consideration of the phenomena, independent of any view of that kind. I am, however, certainly glad that I arrive at this conclusion, because I conceive that any other opinion would lead you to prosecute your anatomical studies with greater anxiety.

I should also say that there is not the fear of communicating the peculiar disease to yourselves, in dissections, of which the persons may have died. Although the venereal disease is capable of communicating infection during life, we do not know of its communicating any noxious effect to the body by dissection after death. With respect to cancer, fungus hæmatodes, and all that class of complaints, we have no knowledge of any effect communicated to the human body from the dissection of persons laboring under such affections. I mentioned to you, that in my own person I only once experienced any inconvenience from a wound contracted in dissection, and that was in opening the body of a person who had died from cancer of the stomach. Now in that case, it happened that the patient was hardly cold when I wounded my fore-finger in sewing up the body; and a very considerable swelling of the axillary glands came on, with great induration. One of my medical friends made a long face, and I found that he conceived that the glands of the axilla had taken on a scirrhous character, in consequence of the disease in the stomach of the patient I had been examining. He mentioned this idea to another gentleman who was with me, under an injunction not to mention it to me, lest it should alarm me. However, this injunction was not observed, and we had a hearty laugh over it. I had no idea of danger, and there was no ground for apprehension.

I may state, that in examining patients who have died from fungus hæmatodes, scirrhus, or the venereal disease, I do not know of any poisonous principle communicated to wounds received on such occasions. There may, however, perhaps be some exceptions to this general observation. There are some instances recorded, of individuals who have received wounds, either in the examination of animals dying under particular states of disease, or in administering during life to these animals; for instance, to glandered horses. There are instances of individuals who have received wounds upon their hands, under such circumstances, in whom a particular train of symptoms has arisen, one circumstance of which has been the formation of abscesses upon various parts of the body. It has been found that the matter of such an abscess has been capable of communicating to other animals—that is, to horses, or asses, the glanders: and there appears to be a possibility of conveying, from such a wound, the malevolence of the peculiar poisonous principle to the human frame."

We know Mr. Lawrence to be too liberal, and too far above the fear of criticism, to dislike a fair and free examination of his doctrines or precepts. In no other spirit do we approach the subject of this distinguished surgeon's lecture.

It will be observed that the first example which the lecturer adduces is that of a young gentleman—"*a robust and hearty person*"—who received so slight a puncture, while sewing up the body of a female who died of peritoneal disease, that he knew nothing of the matter till he was roused from his sleep by "severe pain in the part." Now, as Mr. Lawrence refers the bad effects of these dissection-wounds to "mere local inflammation," in people out of health, there surely is requisite a considerable stretch of the imagination to catenate the severe symptoms which followed in this case, with an injury which was absolutely undiscovered till the patient awoke in the night, from the severity of the pain! Do we see "robust and hearty persons of full habit" suffer, in this manner, when pricked by needles, or when cut by instruments, in the common avocation of life? certainly not. And is not this a strong indication, nay as good a proof as we can expect in medical matters, that a poisonous principle is instilled into dissection-wounds, which modifies and exasperates their character. In the second instance (which we should suppose was the case of Dr. Pett) the dissection was that of a female who also had died of peritoneal inflammation. The puncture by the needle was so slight that the operator knew nothing of the matter, till, in the middle of dinner, the same evening, his attention was arrested by a stinging heat and uneasiness in the finger. In four or five days he was a corpse! Again we ask, would this have been the case had he pricked his finger while sewing a button on his shirt collar?—we suspect that it would not. The practice grounded on the supposition that these cases are merely instances of local inflammation, and the constitutional symptoms being nothing different from those arising from common wounds, is, in our humble opinion, very dangerous. It leads to a system of active depletion which is rarely successful, and often highly destructive. Our brethren will probably ponder on these things before they adopt such vigorous proceedings. Mr. Lawrence acknowledges, and we entirely agree with him, that these accidents rarely occur unless the general health is in a state of derangement. Is not this the case where people are exposed to many other poisons, as malaria for example? But even this very circumstance shews that we have to do with constitutions not in a state of vigour, and consequently not capable of bearing vigorous depletion.

